# Population modification of Anopheline species to control malaria transmission

**DOI:** 10.1080/20477724.2018.1427192

**Published:** 2018-02-01

**Authors:** Rebeca Carballar-Lejarazú, Anthony A. James

**Affiliations:** a Department of Microbiology & Molecular Genetics, University of California, Irvine, CA, USA; b Department of Molecular Biology & Biochemistry, University of California, Irvine, CA, USA

**Keywords:** Population replacement, population alteration, genetically-engineered mosquitoes, gene drive, anti-parasite effector genes

## Abstract

Vector control strategies based on population modification of Anopheline mosquitoes may have a significant role in the malaria eradication agenda. They could consolidate elimination gains by providing barriers to the reintroduction of parasites and competent vectors, and allow resources to be allocated to new control sites while maintaining treated areas free of malaria. Synthetic biological approaches are being used to generate transgenic mosquitoes for population modification. Proofs-of-principle exist for mosquito transgenesis, the construction of anti-parasite effector genes and gene-drive systems for rapidly introgressing beneficial genes into wild populations. Key challenges now are to develop field-ready strains of mosquitoes that incorporate features that maximize safety and efficacy, and specify pathways from discovery to development. We propose three pathways and a framework for target product profiles that maximize safety and efficacy while meeting the demands of the complexity of malaria transmission, and the regulatory and social diversity of potential end-users and stakeholders.

## Introduction

Human disease and deaths resulting from malaria have decreased significantly over the last two decades fueling the enthusiasm for pursuing the goal of malaria eradication [1]. These reductions have been achieved mostly through the increased availability of effective therapeutic drug combinations and insecticide-treated bed nets and indoor spraying [2]. However, there are still over 400 thousand deaths every year, and modeling predicts that the downward trend will reverse with the failure of existing technologies and the absence of new disease-control tools [3].

Vector biologists have proposed a variety of new approaches to contribute to reducing parasite transmission and some are actively pursuing genetic strategies that could impact mosquito populations [3]. These approaches seek either to eliminate or reduce vector mosquito populations below thresholds needed for stable parasite transmission (population suppression), or make them incapable of transmitting pathogens (population replacement/modification) [4]. Proofs-of-principle for both approaches have been demonstrated and efforts are underway to move the advances from the laboratory to the field. A case was made recently for the value of population modification in the eradication agenda and this was based on the demonstrated need for technologies that could provide long-term, cost-effective and sustainable regional malaria elimination [5]. Recent modeling provides additional support for the potential impact of the approaches [6,7]. We complement these reports by reviewing briefly the concept of population modification and posing questions about the challenges of the next steps. A critical task is to specify pathways for further development of the technologies and to define the specific products to be used in population modification control programs.

### What is population modification?

Core concepts of population modification for vector-borne disease control were first aggregated in a single paragraph by Christopher Curtis in which he stated:

“Mutant genes can be imagined the presence of which in a population would be favorable to man, without being very deleterious to the insect. As an example, genes will be considered of a type already known, which make mosquitoes non-infectible by pathogens …. At fixation, the population would be harmless to man …. This type of procedure may be preferable to methods which eradicate a pest species in an area, leaving its ecological niche vacant for reinfestation by immigrants.” [8]

The publication was addressing the theoretical use of chromosome translocations to introgress ‘mutant genes’ into wild mosquito populations, so while defining anti-parasite effector genes, Curtis was at the same time proposing a mechanism to achieve gene drive. We recognize the translocation system as a variant of what is now designated ‘underdominance’. Curtis’ proposal was refined later into a set of operational hypotheses to the effect that (1) the introduction into a population of vector insects of a gene that confers resistance to a pathogen should lead to a decrease in transmission of that pathogen and (2) implicit in this hypothesis is that less transmission will result in less morbidity and mortality [9].

Curtis originally called the approach ‘population replacement’ but this was interpreted by some to mean that mosquitoes developed in the laboratory would be switched, one-for-one, for those existing in the wild. ‘Population modification’ was thought to convey more accurately the goals of the strategies and has been used extensively. More recently, opinions were expressed that calling it ‘modification’ brought with it the negative connotations of the broader use of the phrase ‘genetically-modified organisms’ (GMOs), and ‘population alteration’ was adopted as a less charged description [10]. We use modification in this contribution, but it is important to emphasize that population replacement, modification and alteration all refer to the same category of technologies. World Health Organization (WHO) documents adhere to the original descriptor, population replacement [11].

### What are the benefits of population modification?

Population modification programs alone are not likely to eradicate malaria; this will be achieved using the full array of tools that eliminate parasites in humans (prophylactic and therapeutic drugs, vaccines [when available]) and those targeting the vector (source reduction, insecticides, spatial repellants, and others). However, population modification is expected to provide sustainability to the WHO-defined control, pre-elimination, elimination and prevention-of-reintroduction phases of local malaria elimination [5,6]. This sustainability feature is important because it is the basis for claims that population modification is expected to provide a cost-effective means of preserving local elimination. Population modification strains can serve as barriers to the reinvasion of wild, parasite-susceptible mosquitoes as any insect of the same species introduced into the treated area will acquire parasite-resistance genes through breeding with the local-introduced pathogen-resistant insects, and immigrating parasite-infected people will not be able to infect the resident vectors, and therefore not be a source of parasites for infection of other people in that region.

Population modification also shares with other genetic control strategies the ability of male mosquitoes to find females, and this should facilitate access to vector populations that are unreachable using conventional tools [12]. Population modification strategies can be used in the control phase of an elimination campaign alongside other measures to reduce disease incidence. As the program proceeds, population modification is predicted in models to take on a larger role in the prevention-of-reintroduction phase [5,6]. Parasite-resistant mosquitoes would facilitate consolidation of elimination and allow resources to be allocated to another target region with the confidence that the malaria-free area will remain so. Furthermore, as Curtis proposed, unlike population suppression, population modification is expected to provide a level of environmental safety because it would not result in an ‘empty’ ecological niche that could be occupied by an opportunistic invasive species that may have an expanded vector competence and vectorial capacity. (Vector competence is defined as the intrinsic ability of the insect to transmit a pathogen and includes genetic components [13]. Vectorial capacity is an expression of the efficiency of parasite transmission, and recent formulations include vector competence as a parameter [14]). In addition, modeling supports the conclusion that modification approaches should be stable in low mosquito population densities [6]. This is important in regions where mosquito population sizes fluctuate annually during wet and dry seasons. Similarly, population modification schemes are robust to locally-isolated wild populations since the modified population surrounding a pocket of unmodified mosquitoes will persist until the modified mosquitoes eventually gain access to it. In contrast, population suppression schemes can bypass such disease-bearing populations and then vanish rapidly from the local environment resulting in repopulation of the region with the original unmodified, disease-competent mosquitoes.

### What is needed to achieve population modification and what has been done so far?

**Figure 1.**
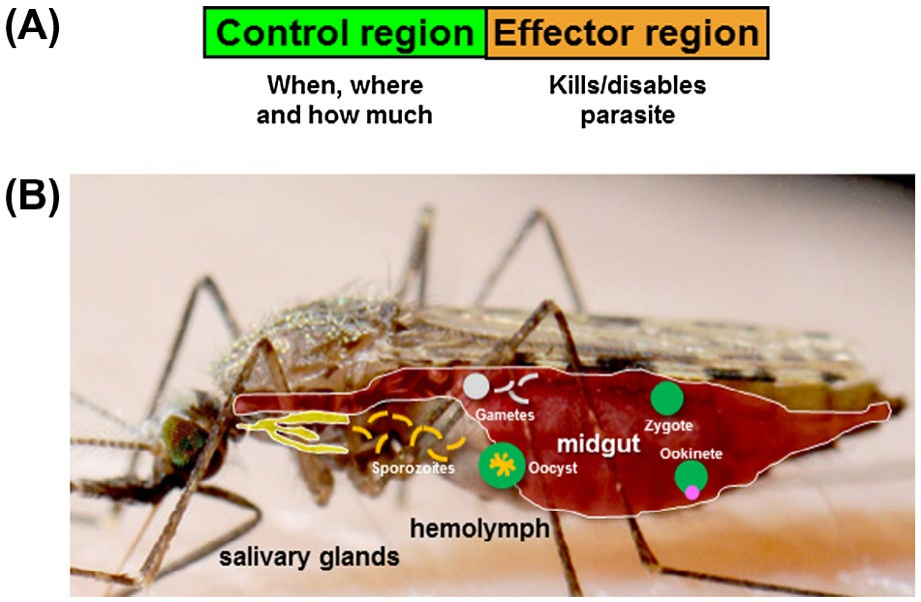
Synthetic approach to making anti-parasite effector genes. A synthetic approach to making an anti-parasite effector gene starts with a simple model of a gene (A) comprising two parts. The *control region* contains *cis*-acting DNA that regulates *when* during development, *where* in the vector insect, and *how much* of a product is made. Constitutive and regulated endogenous mosquito genes with sex-, stage- and tissue-specific expression profiles have been used for the control regions (Table 2). The *effector region* is the expressed portion of the gene that *kills* or *disables the parasite*. This may result from a direct action such as a single-chain antibody that binds the parasite or toxin that kills it, or an indirect action that deprives the parasite of an essential host factor, blocks an important ligand or elevates a systemic immune response (Table 1). (B) Control regions can be selected to deliver effector molecules to specific compartments (*midgut*, *hemolymph* [open circulatory system] and *salivary glands*) in which specific parasite stages are found.

Notes: Malaria parasites enter the mosquito midgut (shaded red) as *gametes* following an infectious blood meal. Male and female gametes fuse to form *zygotes* that mature into motile *ookinetes*. Ookinetes penetrate the midgut epithelium and form an *oocyst* in which thousands of *sporozoites* develop. Sporozoites emerge from the oocyst and migrate through the hemolymph to the salivary glands (shaded yellow) prior to transmission to a new human host.

Research challenges were defined as molecular biologists and geneticists sought to bring Curtis’ theory of population modification to a practical application [9,15,16]. It was important first to show that it would be possible to use molecular genetic tools to engineer genes that would result in parasite-resistance phenotypes in previously competent vector mosquitoes. This laboratory-based phase of the work required the development of synthetic or naturally-derived effector genes that could interfere with parasites in the mosquito and prevent their transmission. Several ingenious approaches were taken and a wide array of effector molecules is now described (Figure 1(A); Table 1). Cis-acting control DNA sequences (promoters, 5′- and 3′-end untranslated regions [UTRs]) of endogenous mosquito genes were identified that allowed the selective expression of the anti-parasite effector molecules in specific compartments of the adult female insects in which the parasites could be found (Figure 1(B); Table 2). Control elements that directed sex- and tissue-specific transgene expression, and were inducible by a bloodmeal proved to be some of the best. Finally, and importantly, transgenesis technologies based on transposable elements were developed that allowed the stable and heritable introduction of engineered genes into Anopheline mosquitoes [17,18]. These combined advances made it possible to demonstrate the proof-of-principle that molecular genetic techniques could be used to make mosquitoes that are incapable of transmitting human malaria parasites [19,20].

**Table 1.** Antimalarial effector genes and targets.

Effector class	Molecule	Target parasite	Reference
Parasite ligands	Pbs21 scFv	*Plasmodium berghei*	[49]
	N2 scFv (targets CSP)	*Plasmodium gallinaceum*	[50]
	scFv 4B7 (targets Pfs25)	*Plasmodium falciparum*	[51]
	scFv 1C3 (targets parasite chitinase)	*P. falciparum*	[51]
	scFv 2A10 (targets CSP)	*P. falciparum*	[51]
	Pbs21 scFv plus Shiva 1	*P. berghei*	[52]
	PfNPNA-1	*P. falciparum*	[53]
Tissue recognition (receptors)	Lectins, Mabs	*P. gallinaceum*	[54]
	SM1 peptide	*P. berghei*	[55]
	Snake phospholipase A2	*P. gallinaceum*, *P. falciparum*	[56]
	Bee phospholipase A2	*P. berghei*	[57]
	Chitinase	*P. gallinaceum*	[58]
Immune response effectors (parasite killing)	Magainins and/or cecropins	Various *Plasmodium* spp.	[59]
	Defensins	*P. gallinaceum*	[60]
	Gambicin	*P. berghei*	[61]
	Gomesin	*P. berghei*	[62]
	Shiva	*P. berghei*	[63]
	NOS	*P. berghei*	[64]
	CEL-III	*P. falciparum*, *P. berghei*	[65]
	TP10	*P. falciparum*	[66]
	AdDLP	*P. berghei*	[67]
	Meucin-25	*P. falciparum*, *P. berghei*	[68]
Antiparasite toxins	Scorpine	*P. berghei*, *P. falciparum*	[69,70]
Others	FREP	*Various Plasmodium spp.*	[71]
	Akt	*P. falciparum*, *P. berghei*	[19]
	Rel2	*P. falciparum*, *P. berghei*	[72]

Abbreviations: AdDLP, *Anaeromyxobacter dehalogenans* defensin-like peptide; Akt, protein kinase B; CEL-III, *Cucumaria echinata* lectin III; CSP, circumsporozoite protein; FREP, fibrinogen-related protein; Mabs, monoclonal antibodies; NOS, nitric oxide synthase; NPNA, repetitive epitope Asn-Pro-Asn-Ala; Pb(s), *Plasmodium berghei* (surface); Pf(s), *Plasmodium falciparum* (surface); Rel, Relish; SM1, salivary gland and midgut peptide 1; scFv, single-chain antibodies; TP, transportan.

**Table 2.** Anopheline mosquito *cis*-acting DNA elements for expressing effector molecules.

Compartment	Gene origin	Expression	Reference
Midgut	Peritrophin	Constitutive	[73]
	Carboxypeptidase	Inducible	[55]
	Trypsin	Inducible	[74]
	G12	Inducible	[74]
Hemolymph	Vitellogenin	Inducible	[75]
Salivary glands	Anopheline antiplatelet protein (AAPP)	Constitutive	[76]
	Apyrase	Constitutive	[77]

**Figure 2.**
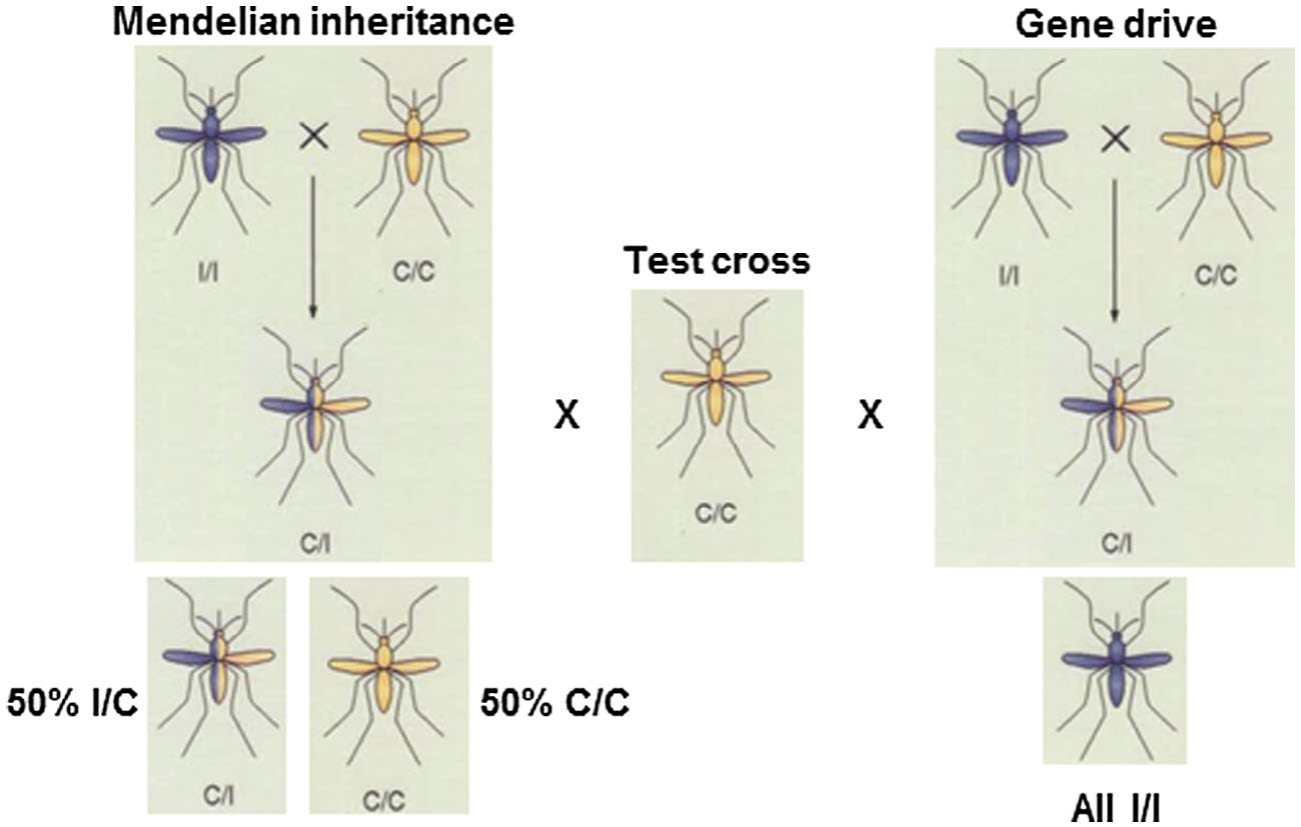
Genotypic and phenotypic outcomes of gene-drive systems.

Notes: Genes-drive systems impact the outcome of inheritance ratios in heterozygous mosquitoes. The scheme shown here is neutral to the drive mechanism (CRISPR/Cas9, transposable elements, underdominance, *etc*.). A wild type mosquito (yellow) is fully competent (C) to transmits malaria parasites and has the genotype C/C. A mosquito carrying a dominant gene (blue) that makes it incompetent (I) to transmit malaria parasites is designated I/I. During Mendelian inheritance (left panels), a cross between the I/I and C/C mosquitoes produces heterozygous progeny, I/C. A test cross of the heterozygotes to the fully competent parental strain, C/C, results in the equal (50%) recovery of progeny in each class, I/C and C/C. In contrast, a similar experiment with a perfect gene-drive system (right panels) results in the recovery of progeny all of which are incompetent, I/I.

The next major challenge was to develop a technology to move synthetic anti-parasite genes into wild populations of mosquitoes in an epidemiologically-relevant time frame [21,22]. Most researchers favor some type of ‘gene-drive’ approach that would rapidly introgress genes by subverting normal patterns of Mendelian inheritance, and the adaption of CRISPR/Cas9 biology, a variation of a homing endonuclease strategy, has great promise (Figure 2) [23–25]. Indeed, this approach comes with such high expectations that a flurry of research community and agency evaluations and commentaries seek to make sure that regulatory regimens and public understanding of the technology can keep pace with the scientific developments [10,26–28]. Alternately, it was shown in laboratory cage trials that releases of population modification strains lacking active drive systems, but expressing mosquito immune system genes altered the midgut microbiome and enhanced transgene spread [29]. This phenomenon could have a positive additive effect on the rate at which these specific constructs introgress into wild populations.

The final and critical challenge is to determine how the technologies can be applied in diverse malaria transmission settings. It is easy to appreciate that small, isolated communities in sub-Saharan Africa differ significantly from villages arrayed along the major rivers in South America, and that neither of these shares much ecologically or demographically with large cities in the Indian subcontinent. However, they all have either or both *Plasmodium falciparum* and *P. vivax* malaria and competent Anopheline vector species. Population modification approaches must be adapted to local conditions and therefore need to consider the biology of the mosquito species and target parasites in these areas. This is likely to be complicated in areas where multiple vector and parasite species contribute to the local malaria burden. Here combining the approach with other technologies, including genetic and conventional population suppression, could have a synergistic effect. In summary, we have excellent proofs-of-principle for generating anti-parasite transgenes and promising gene-drive systems, but much work needs to be done to adapt a population modification approach to a specific malaria transmission setting.

### What are possible pathways from discovery to development of population modification?

**Figure 3.**
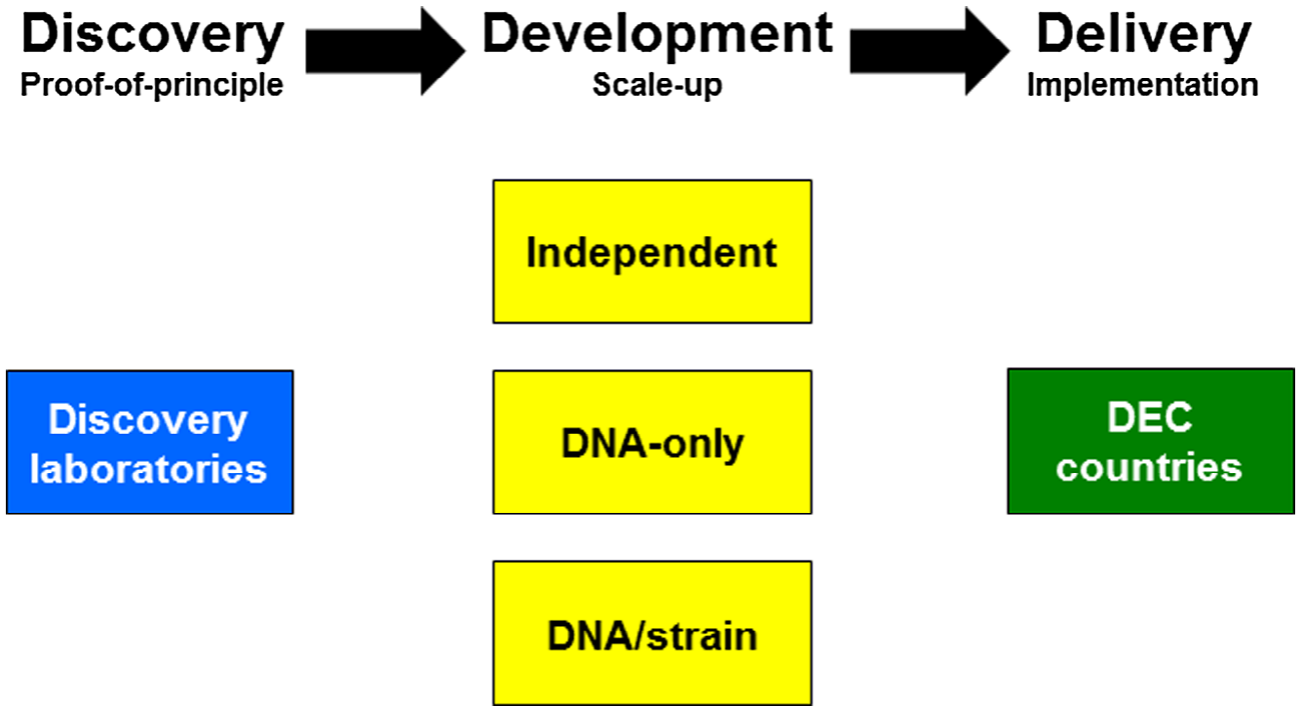
Pathways for development of population modification technologies.

Notes: Population modification technologies will progress from the laboratory-based work of *Discovery* to *Development* before they can be certified for *Delivery*. Discovery laboratories may interact through one or more pathways to bring the technologies to countries with different scientific, regulatory and social capabilities. *Independent* disease-endemic countries (DECs) need little or no assistance from discovery laboratories as published information is sufficient for them to make their own mosquito strains and test them. Some DECS will need help making DNA constructs (*DNA*-*only*) but all subsequent work (transgenesis, phase trials) [11] will take place in-country. Other DECs will need discovery laboratory assistance with making both the DNA constructs and strains to be tested (*DNA/Strain*).

A new technology matures through three stages, discovery, development and delivery, before it is accepted as an established practice (Figure 3). In the context used here, discovery starts with the conception of the idea and progresses through demonstrations of proof-of-principle. Development then investigates the practicality of the discovery to have a real-world impact and often involves scale-up parameters. Finally, delivery encompasses all that would be needed for implementation. Most of the discovery work researching population modification strategies has been done in laboratories in the developed world (designated here as ‘discovery laboratories’) with the intent to apply them in malaria transmission regions in disease-endemic countries (DECs). Several pathways can be imagined for moving the technology to development stages, and these vary in the nature of the product provided by discovery laboratories to end-users and stakeholders. The conceptual formulations and transgene designs of genetics-based population modification strategies and strains are part of the public literature and are accessible in large part to potential users. DECs with the scientific capabilities and public health and regulatory infrastructures (designated here as ‘independent’), for example, some in Asia and Latin America, have the capacity now to use these formulations and designs to generate their own population modification strains that are relevant to their local mosquito species and parasites. From this perspective, the ‘product’ provided by discovery laboratories is the conceptual framework and is available already. There may be a need for discovery laboratories to contribute to training scientists as the independent DEC countries ramp up the technologies, but there is likely to be little demand for discovery laboratory scientists to be involved directly in the deployment of the approaches. Independent DECs also will have the capacity to submit the products for regulatory review, garner community support and raise funds for trials. They will be able to conduct in-country trials and follow through with delivery should the products meet performance criteria and gain regulatory and community approval.

In contrast, DECs lacking some components of the scientific expertise may work more closely with discovery laboratories to adapt population-modification technologies to their local situation. We will discuss two such pathways, one in which the discovery laboratories provide a DNA construct capable of gene drive and conferring a malaria resistance phenotype to a specific Anopheline species mosquito (‘DNA only’; Figure 3), and one in which the discovery laboratories provide the actual genetically-engineered strains for field testing (‘DNA and strain’).

All three of the pathways likely will adhere to a phase-based testing regimen for the technologies, and the vector biology community and we endorse this approach [11,27]. The WHO recognizes four phases: Phase 1 tests comprise the discovery stages and are physically-confined studies in laboratories and insectaries; Phase 2 trials move the products to development and are carried out in small-scale physically and/or ecologically contained field tests; Phase 3 continues development in a series of open release trials that increase in size, length and complexity at one or more sites; and Phase 4 moves the technology to wider application as a malaria control tool in the delivery stage. Specific products are evaluated and subjected to rigorous ‘go/no go’ criteria in each phase. Each step also is accompanied by a phase-specific regulatory structure and includes community engagement and communication activities [5]. These Phases are being reviewed and revised now because of recent successes of gene-drive components.

It is essential that the first field trial of population modification technologies be a success [5]. Funding agencies are unlikely to continue to support the development phases if the first effort ends in failure. Towards that end, site-selection criteria have been proposed to maximize the probability of a positive outcome [30]. Follow-up trials will evaluate product performance in regions with variable levels of parasite transmission, different vector species and seasonal variations in population sizes [11].

### What are the population modification products and how good do they have to be?

While the pathways for further development of population modification approaches may be different for specific DEC countries, there is a clear need for all to define specifically what the final products will be. Furthermore, while we can describe the properties of a perfect population modification product, practical applications require that we determine just how good it must be to be useful. Efforts are now underway to develop Target Product Profiles (TPP) for these genetic technologies. TPPs are used widely in other product-driven disciplines to provide the basis for evaluation of whether a product should be made available for use.

At their most basic level, TPPs have three components, (1) a list of key parameters of the product, (2) a description of each of these features in their perfect form (ideal characteristics), and importantly, (3) a parallel list of how good each feature must be for the product to have practical value (minimally essential). While the ideal characteristics can be formulated by providing what would be the perfect outcome, minimally essential features should be based on empirical studies, or in their absence, rigorous modeling. Population modification TPPs will need to meet efficacy and safety standards and the demands of the different regulatory and cultural contexts in which the products will be used. TPPs also should consider adverse effects and inform product design efforts to mitigate undesired outcomes. Independent DECs likely will develop their own TPPs, but others may work with the discovery laboratories to fashion theirs.

#### A model of a DNA construct-specific TPP

A strong argument for DNA constructs being the products delivered by discovery laboratories is that this approach engages and empowers DEC scientists and stakeholders early in product development, and facilitates the introduction of constructs into local trial-site mosquito species and populations. All phase trials would be conducted by DEC scientists and supervised by in-country authorities. In addition, international regulatory procedures are more favorable for shipping DNA plasmids to a potential trial country than live genetically-engineered strains. Furthermore, the logistics of shipment are expected to be less expensive. This pathway requires that the DEC scientists have the capability to create transgenic mosquitoes. It is crucial that the discovery laboratories contribute to capacity building by training of these scientists in these technologies.

Key parameters of a population modification DNA construct include those that address its fundamental architecture, the anti-parasite effector genes and the autonomous gene-drive system (Table 3). The source DNA parameter could address both scientific and social criteria. Ideally, DNA derived only from the target mosquito would insure that the transgenes function well in the context of the specific species and mitigate concerns about the introduction of ‘foreign’ DNA into a species. However, many components of the final construct may be exogenous and have no corresponding endogenous equivalent. For example, several anti-effector molecules originate in bacteria or vertebrates, or are completely synthetic. While codon-biases can be manipulated to increase expression properties and make them appear more ‘mosquito-like’ to the insect host expression machinery, they are not truly mosquito genes [20]. Furthermore, the elements of CRISPR/Cas9-based gene-drive systems are derived from bacteria and produced synthetically, and the fluorescent marker genes also are exogenous [24,25]. Therefore, it is minimally essential that the source DNA, including the control DNA sequences for expressing the various components, is functional and stable within the target species.

**Table 3.** Draft framework for a target product profile of transgene constructs: Partial list of key parameters and ideal and minimally essential performance features.

Parameter	Ideal	Minimally Essential
Source DNA	All orthologous from target species	All control DNA functional and stable
Stability	No breakdown due to mutation or recombination	Rate of breakdown does not preclude use in local elimination
Size	Small as possible for efficacy	Same as ideal
Complexity	Fewest components as possible to assure efficacy and safety	Same as ideal
Effector molecule species targeting	Kills or disables all human malaria parasites	Kills or disables either *P. falciparum* or *P. vivax*
Effector molecule efficacy	Kills or disables all global populations of human malaria parasites	Kills or disables regional populations of either *P. falciparum* and *P. vivax*
Effector molecule efficiency	Kills or disable all parasites in infected mosquito	Same as ideal
Drive target locus	Highly conserved sequence in gene critical for parasite development, a mutation in which imposes no genetic load on the mosquito	To be determined by target and load properties
Off-target drive effects	No off-target effects	To be determined by target and load properties
Impact of resistant drive targets	No resistant targets	No impact on effector efficacy
Non-target drive effects	No non-target effects	Non-functional in other species
Drive inheritance	Both sexes	One

Stability of the construct refers to the molecular integrity of the DNA conferring the parasite-resistance and drive capabilities. While ideally, there would be no loss of function associated with mutations of the component genes or genomic target sites, or loss through uncoupling by recombination of the drive mechanism from the effector genes, the construct need only last long enough to contribute to maintaining local malaria elimination [6,7]. Basic engineering principles dictate that the smaller and less complex a system is, the less likely it is to fail; however, this may not translate well to biological systems, which can be redundant. Some redundancy may be necessary (for example, multiplexing guide RNAs [gRNAs] and target sites for the gene drive machinery) [31], but it would be good to have the minimally-essential criteria strive to keep the construct as simple as possible to decrease the probability of random mutations while ensuring that it is functionally stable. Simple constructs also are less likely to give rise to unforeseen failures.

The ideal design characteristics of an anti-parasite effector molecule would be that it could disable all world-wide populations of human malaria parasites. This would result from effector molecule targets that are functionally, and possibly structurally, conserved across parasite species so that a single molecular configuration would have the same effect on all of them. While the search for these is on-going, it should be minimally acceptable to have effector molecule(s) that work regionally to disable either *P. falciparum* or *P. vivax* as this would have the greatest impact on morbidity and mortality.

Work that developed single-chain antibodies as effector molecules showed that single copies of two transgenes (‘dual-approach’) could prevent 100% of the sporozoites from entering the salivary glands at infection levels encountered in natural condition [20]. However, laboratory-induced infections resulted in intensities of infection (number of parasites per infected mosquito) much higher than those found in the field and some parasites escaped the effectors and infected the salivary glands. While this hyper-infection could be mitigated by providing additional bloodmeals that increased the levels of effector gene product, it would be prudent to avoid deploying effector genes that in principle might select for increased virulence manifest as higher mean intensities of infection in mosquitoes. This evolutionary route of parasite escape from the effectors could be blocked by adding additional genes to the construct, seeking improvements in the ones already tested, or potentially targeting host factors required for propagation of the pathogen.

The ideal target of the drive construct would be a location in the genome that is highly-conserved in its nucleotide sequence so as to have a low level of naturally-occurring alleles resistant to gRNA-directed cleavage, be in a locus that when mutated by the transgene insertion has a deleterious effect on parasite development, and does not confer a load (fitness cost) to the mosquito. The probability of finding such a site is likely to be low, so the minimally-acceptable should be determined by the efficacy of the DNA construct for the drive and anti-parasite phenotypes. This parameter is addressed more fully once the construct is integrated into its target mosquito host. Also, targeting of host factors essential for parasite development could be accomplished from a benign genomic insertion site by incorporating guide RNAs targeting the desired host factor in the gene-drive construct.

There is considerable debate about the significance of off-target effects (a direct mutational impact in the transgenic organism on a DNA sequence other than the target) in the broader application of CRISPR/Cas9-based technologies, but most of this is associated with efforts to modify specific genes in cells [32]. In this context, an off-target effect would fail to modify the intended gene and may cause a deleterious mutation as a result of gRNA-directed nuclease cleavage at a secondary genomic site. Off-target effects in population modification applications refer to the insertion of the drive-construct into a site in the genome other than that specified by the design of the gRNA or a non-specific mutation event at that site without integration. In the former case, the individual insect would still carry the anti-parasite effector genes and not be able to transmit malaria. Furthermore, it is not likely that this individual insect would be able to sustain drive at the off-target site as the gRNAs would still be specific for the original target site. It is possible that a drive construct could act as a trans-acting mutagen of secondary sites, and the impact would be manifest from the resulting phenotypes. If they are lethal, it should not present a significant problem because the insect carrying it would be removed from the population, but it is possible that other weaker phenotypes may become evident. Phenotypes such as these, and those that reduce mosquito fitness below crucial thresholds or allow them to transmit other pathogens can be monitored in early Phase 1, laboratory-based cages trials once the construct is integrated in the target species, and could be ‘go/no go’ decision points for a specific genomic target site and set of gRNAs.

Natural variation in the DNA of the drive component target site in the wild mosquito population could manifest as loci that are resistant to drive and others may be induced by the drive machinery [7,33,34]. Potential solutions are to use multiple gRNAs (more than one gRNA per target site locale) and multiple target sites in the genome [31]. Here the minimally-acceptable parameter is that these types of variations have no broad impact on the resistance phenotypes in the target mosquito populations. Non-target effects (direct or indirect impact on a species other than the target) can be mitigated by using guide RNAs complementary to DNA present only in the target species, and control DNA that is only functional in the target species (direct), and evaluating the context of an altered mosquitoes in the trial-site environment (indirect). A recent hazard formulation exercise found no basis for any significant indirect effects [10].

Modeling drive inheritance shows that it would be most efficient if it were active in both males and females, however, laboratory-based experiments show that the biology of early development of mosquitoes may favor having drive occur only in males [6,24,35]. This can be achieved using promoters that are functional in the pre-meiotic germ cells of males, although control DNA that allows Cas9 activity in female pre-meiotic germ cells also could be good. Alternatively, it may be possible to develop unstable and/or regulated forms of Cas9 that do not give rise to non-drive events in the egg.

#### A model of a strain-specific TPP

Many DECs face economic and expertise challenges that could prevent them from adopting population modification strategies. In these circumstances, close collaborations with discovery laboratories and funding agencies are needed. This model of interaction has the discovery laboratories making the transgene construct with both effector and drive elements, and being involved in the characterization, colonization and importation of the desired target mosquito species and strains. This work is followed by insertion of the population modification construct into the target mosquitoes, and the conducting of Phase 1 safety and efficacy trials prior to return of the engineered mosquitoes to the DEC of origin. All remaining Phase trials would be in the DEC. This approach has the benefit of making the technology accessible to some of the places where it is needed the most, but also poses significant challenges. Colonization and laboratory adaptation of wild-caught species can be problematic, although practical approaches for maintaining genetic diversity and mating competitiveness have been developed and used [36]. Furthermore, biodiversity protection statutes may slow the export of critical mosquito species and reciprocally, the global regulatory environment for the movement of GMOs may represent a significant hurdle to returning the strains to the trial-site countries [11]. However, these challenges are not insurmountable, and researchers working in Mexico, Brazil and Burkina Faso obtained the necessary permits to import genetically-engineered mosquitoes [37–39].

The laboratory-developed strain would be introgressed over several generations into recently-colonized local mosquito populations prior to the completion of Phase trials in the DEC site. These are the mosquitoes that would be subject to the key parameter assessment of the TPP. This TPP is necessarily more complex and has multiple categories of parameters including effector efficacy, driver efficacy, fitness, safety, and production and release features (Table 4). This TPP also is appropriate for the DNA-only approach once the transgenes have been integrated into the target mosquitoes at the trial site.

**Table 4.** Draft framework for a target product profile of release strains: Partial list of key parameters and ideal and minimally essential performance features.

Category	Parameter	Ideal	Minimally Essential
Effector efficacy	Efficacy (absence of sporozoites in salivary glands)	No parasites (zero prevalence, zero mean intensity of infection) in salivary glands of mosquitoes carrying single copy of single transgene	No parasites (as defined in ideal) in salivary glands of mosquitoes carrying single copy of more than one transgene (‘dual approach’)
	Parasite targets	All populations of all human malaria parasites	Regional populations of *P. falciparum* or *P. vivax*
	Impact (malaria transmission blocking)	Complete absence of malaria transmission in target area, no parasite-infected mosquitoes	To be determined
Driver efficacy	Efficacy (percent population carrying gene)	100% introgression into target wild populations	≥90%
	Target Specificity	Spreads only into target-mosquito species	Same
	Time to introgression	<1 year	2–8 years
Fitness	Male competitiveness	Equal or more than wild-type	>20% of wild-type
	Female fecundity	Equal or more than wild-type	>20% of wild-type
Safety	Safety (vector competence/vectorial capacity)	Strain does not promote disease/pathogen spread in target/non-target organisms; does not facilitate transmission of novel pathogens	Same
	Safety (remediation)	Susceptible to elimination by insecticides, genetic or chemical recall	Susceptible to elimination by any one or combination of currently-existing control methods
Production and Release	Genetic	Maintained as homozygous strain	Balanced heterozygous strain or outcross at every generation
	Formulation	Adult blood fed females	Adult males and females
	Shelf life	3 weeks	2–3 weeks
	Duration of activity/dosing	~10 years	2–8 years
	Dosing regimen	One release regimen per product lifetime	Depends on migration rates and distance, local population structure, seasonal abundance
	Cost of treatment per breeding site	Cost is well within the annual budget allocation for local vector control/public health	Cost does not exceed the standard budget allocation to the local vector control/public health department during a typical malaria epidemic
	Preparation time	Short enough to meet the demand of treatment for large cities	Will not exceed the demand for treatment
	Batch preparation volume/size	1,000s to 100,000s of adult insects needed per treatment per site of target population	To be determined
	Susceptibility to loss of efficacy due to acquired resistance or loss of linkage.	None	Loss of linkage below critical threshold in ≥3 years
	Timing of release	Anytime during the year	Time that maximizes efficacy of introgression (seasonal)
	Release ratios	<1 transgenic:1 wild-type	10 transgenic:1 wild-type

Effector efficacy addresses the real-world malaria transmission dynamics facing a population modification strategy. There are few empirical studies that report the minimally infectious parasite inoculum needed to cause human malaria, but the conclusion appears to be that one or a small number of parasites is all that is needed to establish an infection [40]. These findings support the conclusion that the effector phenotypes should result in no parasites in mosquito salivary glands, and here the ideal and minimally-essential definitions are the same [41]. Modeling studies assume this level of efficiency [6].

The minimally-essential effector gene specificity for parasite targets overlaps that of the construct and would require an effect on either *P. falciparum* or *P. vivax.* The ideal proportion of the mosquito population carrying the effector gene needed to have an epidemiological impact on malaria transmission is 100%. However, in analogy with vaccine coverage, modeling supports the value of population modification approaches that reduce the basic reproductive rate (R_o_) of malaria below one (<1) without having to achieve zero, but this is dependent on the mosquitoes carrying the effector genes having zero parasites in their salivary glands [6].

Criteria for the gene-drive components also reflect the real-world considerations of strain development and deployment. Ideal performance features would be a 100% introgression into the target wild mosquito populations within a single malaria transmission season (<1 year; 10–18 mosquito generations [7]). However, while it could be possible to achieve this ideal, the modeled levels of introgression and speed show favorable epidemiological impacts at 2–8 years [6,7].

Fitness criteria address male mating competitiveness and female fecundity. Previous applications of radiation-based sterile insect techniques (SIT) tolerated reductions of 80% of male competitiveness because this could be overcome by inundative release ratios of 10 treated insects for every wild insect (release ratio = 10:1), and similar effects were predicted from modeling genetically-engineered mosquito strains in which the release ratio are able to compensate for the aggregate fitness losses [42,43]. Furthermore, gene-drive systems are predicted to compensate for some reduction in male competitiveness. This level of male competitiveness also is predicted to be sufficient in mosquitoes if males can meet minimal parameters for local migration frequencies and distances [6]. Similar modeling shows that females also may be able to bear reductions in fecundity as large as 20% and still achieve introgression of the driven genes. Modeling of the impact of a construct that results in an effect on both males and females is needed once each has been determined, and we expect to develop robust strains that perform well beyond these parameter values.

Safety parameters pose several interesting challenges. These include mitigating known possible adverse effects, and having plans to respond to unanticipated events. Carefully planned and executed experiments during Phase trials should ease concerns about whether the transgenes and drive systems could broaden the vector competence or increase the vectorial capacity of the engineered mosquitoes for other pathogens. However, the need and capability to recall the mosquitoes from the field after release is still being debated [27,44]. We suggest that any early open-release trial should be conducted with the opportunity to execute an existing mitigation strategy if needed. For example, insecticide-based protocols could be used to remove engineered mosquitoes from the local environment. However, the practicality of this is questionable in regions where the local insects exhibit some level of insecticide resistance. Indeed, it may become impossible soon to find any mosquito populations anywhere in the malaria-endemic world that do not have some level of resistance to the most commonly-used insecticides. In these circumstances, it may be possible to take advantage of seasonal constraints on population sizes during which low density populations at the end of the rainy season may be easier to eliminate.

The recent enthusiasm and concerns over gene-drive technologies have promoted investigations of genetic- and chemical-based control approaches that could mitigate unwanted outcomes of accidental or intentional release [45]. Significant resources have been made available to develop and test these approaches, but many remain at least as experimental as the original proposed drives. Whether they will be available, or necessary, as field trials start is still to be determined. Again, adding complexity to the genetic make-up increases the risk of unforeseen failures and we urge caution in deploying any so-called reversal-drive systems unless there is a compelling need.

TPP parameters key to later stages of Phase testing include those relevant to scale-up, deployment and costs. One of the more interesting challenges will be defining the life stage of the mosquitoes to be released. Previous opinions were that any release should be done with males only because females can probe and feed and are a nuisance [46]. However, recent deployments of *Wolbachia*-infected strains of dengue vectors for population modification require releasing females as well because the symbiont is inherited maternally [47]. Adults mosquitoes were released in early sterile insect technique (SIT) trials because they have favorable weight-to-number ratios (no need to store or transport in water) and disperse immediately on release [42,48]. In addition, a release of a single blood-fed, fertilized female could be the equivalent of a delayed release of 100–300 mosquitoes [35]. While adult mosquitoes are more fragile than the sub-adult stages and may suffer losses during packaging and transport, releases of mixed populations of adult males and blood-fed females is likely the best approach. Adults can be expected to survive for 2–3 weeks in production facilities prior to release, but it is necessary to know the daily survival rates of the modification strains, and all factors considered, it would be best to release them as soon as the females have been mated and blood fed.

The ideal for the impact of a release would be that a single application (release) should show an effect for the time needed to ensure that there are no human reservoirs of the parasites that can serve as a source of infection of the mosquitoes. Minimally acceptable time frames would be those preventing the reintroduction of parasites into a malaria-free zone. Models support regimens of a single release at multiple sites in a target region to achieve quick introgression of transgenes and outpace drive-based generation of resistant genome target sites [6,7].

Modeling and other analyses support seasonal releases of the population modifications strain [5,6]. Releasing insects at the end of the dry season when wild target populations are small optimizes the release ratios and increases the probably of introgressing the effector genes more rapidly. Increases in mosquito abundance following the onset of rain would amplify the effects of the release strains. Release ratios will depend on the specific drive constructs and can be tested in Phase 1 and 2 cage trials.

TPP parameters for production are critical. Technologies may prove to be robust during the proof-of-principle development, and perform well when scaled up for a field trial. However, to be of practical use, it is necessary to rear the mosquitoes in sufficient numbers and in a time frame needed to meet the demands of a local control protocol. This demand could be for a few thousand mosquitoes for a small village up to hundreds of thousands needed for a complex urban environment. One of the predicted benefits of gene drive-based population modification approaches is that they should reduce the number of mosquitoes needed for releases [5]. It would be ideal if the production mosquitoes could be maintained as stable homozygous strains. This would minimize the amount of outcrossing needed just before production of large numbers for release. Stability is important so that strains do not lose their properties during production. This can be mitigated by scheduled testing of the strains in facilities. The currently available technologies are amenable to the easy remaking of the needed strains should they show any genetic breakdown during maintenance.

## Concluding remarks

The phrase ‘never let the perfect become the enemy of the good’ applies to the transitional period we are experiencing for population modification strategies. There is a need to balance the search for a perfect strain with the demand for something that can be used in the field. TPPs are part of the process that guides the design and development of field-ready tools and will be used during the phase trial evaluations that are to be done prior to advancing a product to the field. We offer the comments here to stimulate the research and stakeholder communities to develop consensus frameworks for TPPs.

## Disclosure statement

No potential conflict of interest was reported by the authors.

## Funding

Research in the James laboratory is supported by awards from the Tata Institute for Genetics and Society, the Bill and Melinda Gates Foundation, the National Institutes of Health [grant number AI29746], the WM Keck Foundation and the University of California, Irvine Malaria Initiative.
